# Supplementary material for: Decreased Muscle-to-Fat Mass Ratio Is Associated with Low Muscular Fitness and High Alanine Aminotransferase in Children and Adolescent Boys in Organized Sports Clubs
Source: J Clin Med. 2021 May 24;10(11):2272. doi: 10.3390/jcm10112272 (PMC8197240; doi:10.3390/jcm10112272)
Supplement: Supplementary file 1 [file jcm-10-02272-s001.zip › jcm-1206004-supplementary.pdf]

## Supplementary

| | | | |
|---|---|---|---|
| No. | Name | | |
| 1. Age | years old | 2. Sex | Male ・ Female |
| 3-1. Sports event | | | |
| 3-2. Competition history (years) | | | |
| 3-3. Past sports history (events and years) | | | |
| 4. Frequency of exercise and sports (Excluding physical education at school) | | 1. At least 3 days a week 2. 1-2 days a week 3. 1-3 days a month 4. Not at al | |
| 5. Exercise and sports time a day (Excluding physical education at school) | | 1. less than 30 minutes 2. 30 minutes to 1 hour 3. 1 hour to 2 hours 4. 2 hours or more | |
| 6. Breakfast intake | 1. eat every day 2. sometimes miss 3. never eat | | |
| 7. Sleep time a day | 1. less than 6 hours 2. more than 6 hours but less than 8 hours 3. more than 8 hours | | |
| 8. Time spent watching TV a day (including video games) | | 1. less than 1 hour 2. 1 hour to 2 hours 3. 2 hours to 3 hours 4. 3 hours or more | |
| 9. Height, Weight | cm | kg | |

・Please circle the applicable items below and fill in the parentheses.

1. I have been hospitalized before. What kind of illness was it? ( )
2. I have a close relative who died suddenly of an illness under the age of 50. What kind of illness was it? ( )
3. I have had heart disease. What kind of disease was it? ( )
4. I have had an abnormal ECG. What kind of abnormality was it? ( )
5. I have allergies. What kind of allergy was it? ( )
6. I have diabetes. At what age? ( )
7. I have asthma or another lung disease. At what age? ( )
8. I have experienced my pulse disturbed during exercise. ( )
9. I have experienced chest pain or distress during exercise. ( )
10. I have experienced strong palpitations during exercise. ( )
11. I have experienced dizzy or faint during or after exercise. ( )
12. I am very tired after exercise. ( )

・Do you have, or have you ever had bone, joint, or muscle injury?

①No ②Yes

(a) Please circle the location of the injury.

(b) Please describe the intensity of your pain or symptoms.

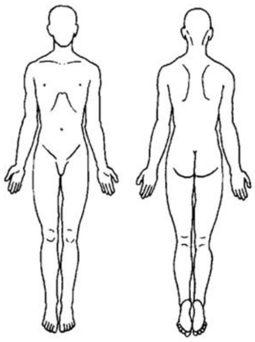

・Please write down any questions you would like to ask the doctor.

**Figure S1.** Preliminary questionnaire.
